# The effects of whole green tea infusion on mouse urinary bladder chemical carcinogenesis

**Published:** 2014-02

**Authors:** Andreia Henriques, Regina Arantes-Rodrigues, Ana I Faustino-Rocha, Catarina I Teixeira-Guedes, Jacinta Pinho-Oliveira, Daniela Talhada, José H Teixeira, Andreia Andrade, Bruno Colaço, Maria N Paiva-Cardoso, Maria J Pires, Ana MVD Ferreira, Fernando M Nunes, Paula A Oliveira

**Affiliations:** 1 Department of Veterinary Sciences, ECAV, University of Trás-os-Montes and Alto Douro, 5001-911, Vila Real, Portugal; 2 Department of Zootechnics, University of Trás-os-Montes and Alto Douro, 911 Vila Real, Portugal; 3 Center for the Study of Animal Sciences (CECA), University of Porto, 4485-661, Porto, Portugal; 4 Chemistry Research Center, CQ-VR, University of Trás-os-Montes and Alto Douro, 5001-801, Vila Real, Portugal; 5 Centre for the Research and Technology of Agro-Environmental and Biological Sciences, University of Trás-os-Montes and Alto Douro, 5001-911, Vila Real, Portugal

**Keywords:** Green tea, Mice, * N*-butyl-*N*-(4-hydroxybutyl)nitro- samine, Urinary bladder cancer

## Abstract

***Objective(s):*** Green tea (GT) is one of the most popular beverages worldwide whose beneficial effects on health have been demonstrated. Recent studies suggest that GT may contribute to reduction of cancer risk and progression. The aim of this study was to evaluate the effects of whole GT on urinary bladder chemical carcinogenesis in male and female ICR mice.

***Materials and Methods:*** The GT characterization was performed using spectrophotometric methods. Urinary bladder lesions were induced using *N*-butyl-*N*-(4-hydroxybutyl) nitrosamine (BBN) by gavage during 10 weeks and whole GT (0.5%) was provided *ad libitum *during 20 weeks.

***Results:*** Animals from groups BBN+GT and BBN only developed preneoplastic lesions.

***Conclusion:*** We did not observe any effects by GT infusion administration on urinary bladder cancer development.

## Introduction

Urinary bladder cancer is one of the most common diseases worldwide. It is more frequent in men than in women, representing the fourth and eighth causes of cancer, respectively (1). In developed countries, there are risk factors associated with urinary bladder cancer development, namely tobacco smoking, chemical carcinogens, and ionizing radiation (2). *Schistosoma haematobium*’s chronic infection is another factor that contributes to increase in the incidence of this disease (3). *N*-butyl-*N*-(4-hydroxybutyl) nitrosamine (BBN) is a chemical carcinogen that in rodents induces the development of preneoplastic and neoplastic urothelial lesions similar to those observed in humans and accompanied by a significant inflammatory reaction (4).

There is a great debate regarding the use of natural and food compounds in cancer chemoprevention (5). Green tea (GT), manufactured from leaves of *Camellia sinensis*, is one of the most popular beverages worldwide (6). GT composition is very complex and contains proteins, carbohydrates, lipids, vitamins, polyphenols, xanthic bases, pigments, and minerals. Major polyphenols present in GT are flavonoids, especially catechins, that have garnered considerable attention due to beneficial effects on health, including antioxidant, anti-inflammatory, and chemopreventive effects (5). Chemopreventive effects of GT polyphenolic catechins on carcinogenesis have been reported in a variety of animal tumor models, including inhibition of forestomach and/or lung carcinogenesis in mice, esophageal, mammary gland and glandular stomach carcinogenesis in rats, and intestinal carcinogenesis in mice and rats (7). All of cited studies were performed with specific GT compounds; none of them evaluated the effect of whole GT during urinary bladder carcinogenesis in male and female ICR mice. Based on these facts, the purpose of this study was to evaluate the chemopreventive effects of whole GT on urinary bladder cancer development induced by BBN in male and female ICR mice.

## Materials and Methods

***Chemical characterization of green tea***

The GT infusion and leaves were used to determine total polyphenols, flavonoids, and antioxidant activities using spectrophotometric methods. To determine the total polyphenols concentration, the Folin-Ciocalteu method was used. Flavonoids content in GT was determined according to the method described by Quettier-Deleu *et al* (8) using an ethanol solution with AlCl_3 _2%. Flavonoid content was calculated as quercetin (Qr) equivalent (milligrams per gram) from a calibration curve. Antioxidant activities of the GT and leaf samples were estimated by trolox equivalent antioxidant capacity assay (TEAC) according to Liebert *et al *(9). The TEAC test was based on the oxidation of 2,2’-azino-bis (3-ethylbenzothiazoline-6-sulfonate) (ABTS) to the radical cation ABTS^·+^. All samples were analyzed in triplicate.

The chemical characterization of GT infusion polyphenols was performed by high performance liquid chromatography (HPLC) using an Ultimate 3000 Column Compartment Dionex equipped with a photodiode array detector (PDA) 100 Dionex. An ACE5C18 reversed phase column (250×4.6 mm×5 µm) was used. The elution (1 ml/min) was performed using a solvent system comprising solvent A (5% of formic acid) and solvent B (methanol), using a gradient starting with 5% of solvent B until 2 min, linearly increasing to 65% of solvent B until 65 min and decreasing to 5% at 65 min and held constant until 75 min, at 35°C. The injection volume was 50 µl. The PDA detector was set in the range of 200-600 nm. Wavelength of 280 nm was used to identify catechins and caffeine and wavelength of 325 nm was used to detect hydroxycinnamic acids. The compounds were identified based on their retention time when commercial standards were available and by comparison with the UV-spectrum. The composition of GT infusion was analyzed at 0, 24, and 48 hr after its preparation.

***Animals***

The design and experimental procedures were performed in accordance with the EU regulations (Directive 2010/63/EU) on protection of animals used for experimental and other scientific purposes. Forty-one, five-week-old ICR mice (21 males and 20 females), were obtained from Harlan-Interfauna (Spain). Animals were placed in polycarbonate cages using wood chips for bedding. During experimental protocol, animals were housed in controlled conditions of temperature (23±2°C), humidity (55±5%), lighting (12/12 hr light/dark cycle), and air system filtration (10–20 ventilations/hr). Standard laboratory diet (Harlan) and GT were provided *ad libitum*.

***Chemicals***

BBN was purchased from Tokyo Kasei Kogyo (Japan). The GT leaves (Thea Sinensis L.) were purchased from Augusto Coutinho Ervanários (Portugal). Folin-Ciocalteu reagent, GA, ABTS, trolox (Tx), Qr were purchased from Sigma (USA).

***Experimental design***

After one week of acclimatization period, animals were randomized in six experimental groups each [*Males* group I: BBN+GT (n=8); group II: BBN (n=7); group III: GT (n=6); *Females* group IV: BBN+GT (n=7); group V: BBN (n=7); group VI: GT (n=6)]. BBN was prepared in a concentration of 0.05% with bidistilled water and absolute alcohol. It was administered to animals by gavage in mean doses of 7.25 mg/mouse, two times per week, during ten consecutive weeks*. *The whole GT (0.5%) was daily prepared and given *ad libitum *to groups I, III, IV and VI for 20 consecutive weeks in dark bottles. The GT was prepared by adding five grams of tea leaves to boiled water and letting it rest for four min. Ponderal homogeneity index (PHI) [PHI=2W_l_/(W_h_+W_l_)_; _W_h_-Higher weight, W_l_-Lower weight], was determined to evaluate the homogeneity between groups. Animals’ initial and final body weights were measured to determine weight gain (WG) (WG=W_f_-W_i_/W_f_x100; W_f_-Final weight, W_i_-Initial weight). The organs’ relative weights were measured as the ratio of the mouse’s organ weight by the mouse’s final weight.

***Sample collection***

Twenty weeks after the start of the experiment all animals were sacrificed by pentobarbital sodium (Eutasil^®^, France) overdose. Complete necropsies were performed after mice macroscopic evaluation. The spleen, liver, kidneys, lungs, and heart were collected and weighed. The mice urinary bladders were collected and weighed according to the technique described by Oliveira *et al* (10). After fixation, the urinary bladders were longitudinally cut to observe macroscopic lesions in their mucosal surface. For the histological study, the urinary bladders and organ samples were embedded in paraffin and sections of 2 µm were cut and stained with hematoxylin and eosin (H&E). Histological lesions found in different groups were classified and staged according to Eble *et al* (11). Microscopic urinary bladder evaluation was categorized as normal urothelium, simple hyperplasia, dysplasia, papilloma, and squamous metaplasia. The number of inflammatory aggregates was counted in each urinary bladder per section; the mean number of inflammatory aggregates was calculated for each group. All morphological readings were double-checked.

**Table 1:** Green tea chemical characterization

Sample (S)	Total phenols		Flavonoids		Antioxidant capacity	
	mg GA/ 100 g of infusion	mg GA/ 100 g of GT leaves	mg Qr/ 100 g of infusion	mg Qr/ 100 g of GT leaves	mg Tx/ 100 g of infusion	mg Tx/ 100 g of GT leaves
S_1_	34.80	69.64	3.53	7.07	193.18	386.36
S_2_	36.40	72.81	2.84	5.69	183.72	367.44
S_3_	34.50	69.03	2.82	5.65	201.92	403.85
M±SD	35.23±1.02	70.49±2.03	3.06±0.40	6.13±0.81	192.94±9.10	385.88±18.2

S- Sample; GA- Gallic acid; Qr- Quercetin; Tx- Trolox; Mean±SD

***Statistical analysis***

All analyses were performed using SPSS (USA). Continuous data were analysed using ANOVA. Pathological examinations were analysed by the chi-square test. Data were expressed as mean±standard deviation (SD). Results were considered statistically significant when *P*<0.05. 

## Results

This study evaluated the effect of oral administration of whole GT on the development of urothelial lesions chemically-induced by BBN in female and male mice. We observed that the concentrations of total phenols and flavonoids, and antioxidant capacity were higher in GT leaves when compared with GT infusion (Table 1). In order to verify if the GT compound concentrations varied over time, the infusion was analyzed by HPLC at 0, 24, and 48 hr after preparation. The resultant chromatograms at 0 and 24 hr showed that the polyphenol concentration of GT decreased 24 hr after its preparation (data not shown).

Throughout the course of the experiment two animals died during BBN oral gavage (one from group II and one from group V). We did not observe any changes in animals’ behavior or clinical signs of distress/discomfort. During the experimental protocol, the groups showed a similar food and water intake. PHI, initial and final weights and WG were similar among groups. We did not find any statistically significant differences in the mean values and relative weights of spleen, liver, kidneys, lungs, heart, and urinary bladder (data not shown).

Males from group III (GT) and females from group VI (GT) did not show any urothelial lesions. In males, the incidence of urothelial lesions was greater in animals from group I (BBN+GT) than animals from group II (BBN). Nevertheless, we observed a lower incidence of simple hyperplasia, squamous metaplasia, and dysplasia in group IV females (BBN+GT) compared with group V (BBN). However, these results were not statistically significant between groups (Table 2). The mean number of inflammatory aggregates was greater in males than in females. Groups I (BBN+GT) and IV (BBN+GT) showed a lower number of inflammatory aggregates compared with groups II (BBN) and V (BBN), respectively (Table 2).

## Discussion

Most research works conducted with GT use only one purified or partially purified component. In this study we evaluated the effect of whole GT infusion in mice experimental urinary bladder cancer induction instead of a particular compound, because this mimics the normal human tea consumption.

In order to characterize the composition of GT, we performed a total phenols, flavonoids, and antioxidant capacity analysis. According to our results, GT leaves had a higher total phenols, flavonoids, and antioxidant capacity than GT infusion; however, approximately 50% of total phenols, flavonoids, and antioxidant capacity remained in the infusion. These results differ from the study conducted by Sato and Matsushima (12) that reports that only 30% of total initial compounds present in GT leaves are available in tea infusion. The infusion used in this study was stored in dark recipients to prevent the photodegradation of its components. However, we could not avoid the degradation over time caused by oxidation. Our HPLC analysis on GT infusion demonstrated that its polyphenol concentration decreased and almost disappeared at 48 hr; we decided to prepare fresh GT infusion every 24 hr. These results are in accordance with Bianchi *et al* (13).

Taking into account that frequent mice manipulation can induce severe distress, we decided to provide GT infusion in bottles* ad libitum*. In this study we chose BBN oral administration by gavage, because the oral route was selected to administrate GT infusion. The animals’ health states were normal during the experiment. The mortality rate observed in this study was associated with oral gavage, and in accordance with Arantes-Rodrigues *et al* (14).

The groups that only drank GT infusion did not have any urothelial lesions. Although in humans the incidence of urinary bladder cancer is greater in men than in women, in this work we observed similar results between male and female mice. According to Miyamoto *et al* male mice are more likely than females to develop urinary bladder cancer induced by BBN (15). Animals from groups exposed to BBN+GT (I and IV) showed a lower mean number of inflammatory aggregates than animals from BBN groups (II and V), probably due to GT anti-inflammatory effects. 

Other studies have demonstrated the antitumor capabilities of GT (7), however the mechanisms involved are not fully understood. 

**Table 2:** Urothelium histopathological evaluation n (%) and inflammatory aggregates (mean±standard deviation)

Groups		Male			Female		
Microscopic analysis		I (n=8) (BBN+GT)	II (n=6) (BBN)	III (n=6) (GT)	IV (n=7) (BBN+GT)	V (n=6) (BBN)	VI (n=6) (GT)
Histological lesions	Normal urothelium	0 (0%)	0 (0%)	6 (100%)	0 (0%)	0 (0%)	6 (100%)
	Simple hyperplasia	6 (75%)	3 (50%)	0 (0%)	3 (42.8%)	4 (66.7%)	0 (0%)
	Dysplasia	7 (87.5%)	5 (83.3%)	0 (0%)	4 (57.1%)	4 (66.7%)	0 (0%)
	Papilloma	2 (25%)	0 (0%)	0 (0%)	0 (0%)	0 (0%)	0 (0%)
	Squamous metaplasia	0 (0%)	0 (0%)	0 (0%)	0 (0%)	2 (16.7%)	0 (0%)
Inflammatory aggregates		3.50±4.46	4.67±2.99^a^	0.42±0.90	3.50±2.56	3.83±3.13^b^	0.25±0.45

^a^ *P* < 0.05 *versus* Group III; ^b ^*P* < 0.05 *versus *Group VI

## Conclusion

Animals from BBN and BBN+GT groups only developed preneoplastic lesions. The number of inflammatory aggregates was lower in animals that drank GT. We can conclude that the whole GT infusion had influence on urothelial inflammation. For future investigations we suggest the use of different GT infusion concentrations.
